# Cervical sagittal alignment is influenced by changes in thoracic and lumbar sagittal alignments after correction surgery in patients with lenke type 6 adolescent idiopathic scoliosis

**DOI:** 10.1016/j.xnsj.2022.100166

**Published:** 2022-09-05

**Authors:** Toshiki Okubo, Mitsuru Yagi, Satoshi Suzuki, Yohei Takahashi, Satoshi Nori, Osahiko Tsuji, Narihito Nagoshi, Morio Matsumoto, Masaya Nakamura, Kota Watanabe

**Affiliations:** a Department of Orthopaedic Surgery, National Hospital Organization, Murayama Medical Center, 2-37-1 Gakuen, Musashimurayama, Tokyo 208-0011, Japan; b Department of Orthopaedic Surgery, Keio University School of Medicine, 35 Shinanomachi, Shinjuku, Tokyo 160-8582, Japan

**Keywords:** Adolescent idiopathic scoliosis, Lenke type 6, Cervical sagittal alignment, Posterior correction and fusion surgery, C2-7 angle, Thoracic kyphosis, T10-L2 kyphosis, Lumbar lordosis

## Abstract

**Background:**

Few studies have examined the changes in cervical sagittal alignment (CSA) and its relationship with other sagittal alignments in AIS patients with major thoracolumbar/lumbar (TL/L) curve who underwent correction surgery. This study investigated the radiographical changes in CSA after correction surgery in patients with Lenke type 6 adolescent idiopathic scoliosis (AIS) and assess any possible factors affecting postoperative CSA.

**Methods:**

Forty-four patients with Lenke type 6 AIS (3 males and 41 females, mean age at surgery of 15.6 ± 2.8 years) who could be followed up for 3 years after correction surgery were included in this study. Variations of outcome variables were analyzed in various spinal sagittal parameters using radiographic outcomes. Univariate correlation analyses were used to evaluate possible factors influencing the postoperative CSA. The Scoliosis Research Society (SRS)-22 questionnaires and the Oswestry Disability Index (ODI) were used for clinical evaluation, and the changes between pre- and 3-year post-operation were compared.

**Results:**

The Cobb angle of the major and minor curve was significantly improved after correction surgery. Furthermore, CSA, such as C2-7 angle and T1 slope, changed significantly postoperatively. The magnitude of coronal curve correction did not affect CSA postoperatively, while the postoperative TK, T10-L2 kyphosis and LL were significantly correlated with the postoperative C2-7 angle, respectively. None of the patients in this study complained of neck or low back pain during the period up 3 years after the operation. Comparing each domain of SRS-22 or ODI score, these were unchanged between pre-, 1-year, and 3-year post-operation, with no statistically significant differences.

**Conclusions:**

CSA changed significantly after correction surgery, and cervical kyphosis indicated a tendency to decrease in Lenke type 6 AIS patients, which was associated with an improvement in thoracic and lumbar sagittal alignment, not correction for coronal deformity.

## Background

Posterior correction and fusion surgery, whose main purpose is to obtain a coronally balanced spine using spinal instrumentation during surgery, is a common surgical procedure for patients with adolescent idiopathic scoliosis (AIS) [1], [2], [3]. However, recently, spinal sagittal alignment, such as the cervical, thoracic, and lumbosacral spine, has been considered a more important subject in AIS [4,5]. Several reports showed that sagittal malalignment could increase the risk of developing neck or back pain and neurological symptoms in later life, suggesting that spinal sagittal alignment may be correlated with clinical and functional outcomes in AIS patients [6,7].

To date, postoperative cervical sagittal alignment (CSA) has been reported to be closely related to thoracic sagittal alignment [8], [9], [10], [11], [12], [13]. However, most of these reports have been evaluated in AIS patients with a major thoracic curve, such as Lenke type 1. In contrast, few studies have evaluated changes in CSA and its relationship with other sagittal alignments after surgery in AIS patients with a major thoracolumbar/lumbar (TL/L) curve, such as Lenke type 6 [14], [15], [16]. Therefore, investigating the changes in each spinal sagittal alignment following surgery and the corresponding factors that influence the postoperative CSA, is essential to clarify the benefits of correction surgery for Lenke type 6 AIS patients.

The purpose of this study was to examine changes in various spinal sagittal parameters 3 years after correction surgery and to evaluate any possible factors that influence CSA in Lenke type 6 AIS.

## Methods

### Study design, patient's demographics, and characteristics

A total of 44 patients with Lenke type 6 AIS (three males [6.8%] and 41 females [93.2%]) who underwent correction surgery with spinal instrumentation at our institute between 2005 and 2017, with ages at surgery ranging from 13 to 20 years (mean age, 15.6 ± 2.8 years), were included in this study. The inclusion criteria were as follows: 1) diagnosed with Lenke type 6 AIS at an age between 12 and 20 years; 2) no previous spine surgery; 3) no neuromuscular disorders, congenital vertebral deformity, trauma, or other pathological conditions; 4) clear visualization of the whole spine on radiographs pre- and postoperatively; and 5) a minimum of 3-year follow-up after surgery. All information on demographics, imaging, and surgical data were retrospectively obtained from patients’ medical records and radiological images.

All surgeries were performed by both or either of two surgeons (K.W. and M.M.) with the same surgical strategy. Fusion levels were selected according to the following strategy during the entire study period: the upper instrumented vertebra (UIV) was chosen as the upper end vertebra (UEV) of the minor curve, while the selection of the lower instrumented vertebra (LIV) was determined as L3 vertebra based on the level of the lower end vertebra (LEV) in most of the cases at the preoperative planning. However, in cases where the L3 vertebra did not touch the central sacrum vertical line (CSVL) on the lateral side bending radiographs, the LIV was determined as L4 vertebra.

In our institute, to correct lumbar vertebral rotation, we pushed the lumbar hump to the ventral side using the cantilever technique from the caudal side. The rod rotation technique was not often performed. Because excessive stress was placed on the L3 vertebra, off-set hooks were sometimes used on the convex side of the L3 vertebra. Moreover, reduction screws were used to pull up the concave side of the minor curve, aiming to form a kyphosis in the thoracic spine as much as possible.

This study was approved by the ethics and institutional review board committee (approval number: 20090042), and all subjects gave their informed consent for inclusion prior to treatment.

### Radiographical data

All patients in the present study underwent a whole-spine radiological evaluation at ‘pre-operation’ and ‘3-year post-operation’. The X-ray findings contained standing erect whole-spine posteroanterior and lateral radiographs. For lateral views, the patients stood with their knees locked, their feet shoulder-width apart, looking straight ahead with their elbows bent and their knuckles in the supraclavicular fossa bilaterally [17,18].

The coronal flexibility of the curves or the primary and compensatory curves was assessed according to supine bending radiographs. Bending radiographs and the level of UEV/LEV have been recommended to help determine the levels to be selected in the correction of scoliosis.

Cobb angles were measured using the above whole-spine X-ray in standing position, and the major and minor curve were measured as described by Lenke *et al* [19]. We also measured various spinal sagittal parameters as follows: sagittal vertical axis (SVA, plumb lines of C7), C2-7 SVA, C2-7 angle, T1 slope, T5-12 thoracic kyphosis (TK), T10-L2 kyphosis, L1-5 lumbar lordosis (LL), pelvic tilt (PT), pelvic incidence (PI), and sacral slope (SS). For C2-7 angle, negative value indicated kyphosis, whereas positive values indicated lordosis. All data were described as mean ± standard error of three independent measurements, and categorical variables were presented as percentages. To reduce inter-observer variations, radiographs were measured independently by two authors of this study.

### Postoperative clinical assessment

The clinical outcome was assessed using the Scoliosis Research Society (SRS)-22 questionnaire and the Oswestry Disability Index (ODI), which were completed before, 1 year, and 3 years after correction surgery.

### Statistical analysis

Changes in various spinal sagittal parameters were compared, respectively, between pre-operation and 3-year post-operation using the Mann-Whitney U test. Clinical outcomes (each domain of SRS-22 and ODI score) were compared between pre-operation, 1-year post-operation, and 3-year post-operation using Kruskal-Wallis test followed by a post hoc test for multiple comparisons. A correlation analysis was performed using a Pearson test. A *P*-value less than 0.05 was considered statistically significant. All statistical analyses were performed using SPSS version 26.0 (SPSS Inc., Chicago, IL). No statistical sample size calculations were conducted in advance of this study. However, post hoc power analysis was performed to indicate their reliability in terms of attained power (0.0 ≤ 1−β ≤ 1.0) using G*Power software (version 3.1.9.2, Heinrich Heine Universität Düsseldorf).

## Results

### Radiographical changes in coronal and sagittal alignment after correction surgery

Patients’ demographics, characteristics, and surgical data are shown in Table 1. The mean number of fixed vertebrae was 9.8 ± 0.9. The UIV was at T3-7 (T3 in two cases, T4 in one, T5 in 27, T6 in 13, and T7 in one), whereas the LIV was at L1-4 (L1 in one case, L2 in two, L3 in 34, and L4 in seven). The most common fused level was located at T5-L3, accounting for 50.0% of all cases (T3-L3 in two cases, T4-L3 in one, T5-L2 in two, T5-L3 in 22, T5-L4 in three, T6-L1 in one, T6-L2 in one, T6-L3 in eight, T6-L4 in three, and T7-L4 in one). The mean surgical time was 150.4 ± 32.5 (90-255) minutes, while the estimated blood loss was 439.8 ± 181.3 (100-920) mL. In addition, there were no complications during 3 years after operation, such as loss of intraoperative monitoring, paralysis, deep wound infection, implant failure, pseudarthrosis, or additional surgeries.

**Table 1:** Demographic and clinical characteristics of all patients.

Case	n = 44
**Age at surgery (y/o)**	15.6 ± 2.8
**Gender, no. (%)**	
Male	3 cases (6.8)
Female	41 cases (93.2)
**No. of fixed vertebra**	9.8 ± 0.9
**UIV, no. (%)**	
T3	2 cases (4.5)
T4	1 case (2.3)
T5	27 cases (61.4)
T6	13 cases (29.5)
T7	1 case (2.3)
**LIV, no. (%)**	
L1	1 case (2.3)
L2	2 cases (4.5)
L3	34 cases (77.3)
L4	7 cases (15.9)
**Fused levels, no. (%)**	
**T3-L3**	2 cases (4.5)
**T4-L3**	1 case (2.3)
**T5-L2**	2 cases (4.5)
**T5-L3**	22 cases (50.0)
**T5-L4**	3 cases (6.8)
**T6-L1**	1 case (2.3)
**T6-L2**	1 case (2.3)
**T6-L3**	8 cases (18.2)
**T6-L4**	3 cases (6.8)
**T7-L4**	1 case (2.3)
**Surgical time, (min)**	150.4 ± 32.5
**Estimated blood loss, (ml)**	439.8 ± 181.3

Values indicate mean ± standard deviation.

UIV, upper instrumented vertebra; LIV, lower instrumented vertebra.

The mean preoperative Cobb angle of the minor curve was 49.8 ± 8.8°, which improved significantly after correction surgery (3-year post-operation, 13.2 ± 6.9°; *p* < 0.001). The mean Cobb angle of the major curve also improved significantly (pre-operation, 57.3 ± 11.9° *vs.* 3-year post-operation, 7.7 ± 7.6°; *p* < 0.001). The mean T10-L2 kyphosis decreased significantly from 10.3 ± 7.1° to 1.4 ± 3.5° (p = 0.019), while LL increased significantly from 30.3 ± 6.2° to 43.5 ± 6.7° (p = 0.011), TK from 7.3 ± 4.3° to 13.9 ± 8.0° (p = 0.024), respectively. Furthermore, the mean C2-7 angle also increased significantly from -18.0 ± 8.6° to -4.5 ± 9.7° (p = 0.003), T1 slope from 8.5 ± 6.1° to 14.1 ± 5.9° (p = 0.018), respectively. On the other hand, the mean values of SVA, C2-7 SVA, spino-pelvic sagittal parameters (PI, PT, SS) did not change significantly after correction surgery (Table 2).

**Table 2:** Comparison of preoperative and 3-year postoperative radiological parameters among all cases.

Parameter	Pre-operation	3-year post-operation	*p* value (Pre- vs 3-year post-)
Cobb angle (minor, °)	49.8 ± 8.8	13.2 ± 6.9	<0.001***
Cobb angle (major, °)	57.3 ± 11.9	7.7 ± 7.6	<0.001***
SVA (C7PL, mm)	-24.9 ± 25.6	-27.9 ± 12.2	0.787
C2-7 SVA (mm)	16.6 ± 7.9	17.5 ± 5.3	0.763
C2-7 angle (°)	-18.0 ± 8.6	-4.5 ± 9.7	0.003**
T1 Slope (°)	8.5 ± 6.1	14.1 ± 5.9	0.018*
TK (°)	7.3 ± 4.3	13.9 ± 8.0	0.024*
T10-L2 kyphosis (°)	10.3 ± 7.1	1.4 ± 3.5	0.019*
LL (°)	30.3 ± 6.2	43.5 ± 6.7	0.011*
PI (°)	50.1 ± 8.9	52.3 ± 7.1	0.824
PT (°)	17.3 ± 6.5	15.4 ± 3.3	0.661
SS (°)	32.2 ± 4.0	34.8 ± 6.7	0.597

Values indicate mean ± standard deviation.

*p < 0.05, **p < 0.01, ***p < 0.001: statistically significant differences.

SVA, sagittal vertical axis; C7PL, plumb lines of C7; TK, thoracic kyphosis; LL, lumbar lordosis; PI, pelvic incidence; PT, pelvic tilt; SS, sacral slope.

We evaluated whether the magnitude of the minor or major curve correction (the amount of change in the Cobb angle of the minor or major curve before and after correction surgery) would influence each radiological spinal sagittal profile at 3 years postoperatively. As a result, there was no statistically significant correlation among them (Table 3).

**Table 3:** Correlation analysis between the magnitude of the minor or major curve correction and each of the 3-year postoperative radiological sagittal parameters.

Postoperative spinal sagittal parameters at the 3-year post-operation	Minor curve		Major curve	
	*r*	*p* value	*r*	*p* value
SVA (C7PL) C2-7 SVA C2-7 angle T1 slope TK T10-L2 kyphosis LL PI PT SS	-0.131 -0.124 -0.245 0.190 -0.108 -0.217 0.165 0.087 -0.093 0.105	0.203 0.291 0.193 0.355 0.541 0.311 0.484 0.837 0.635 0.662	-0.250 -0.259 -0.255 0.172 -0.025 -0.263 0.174 0.025 -0.072 0.076	0.183 0.175 0.181 0.374 0.896 0.291 0.490 0.922 0.776 0.763

SVA, sagittal vertical axis; C7PL, plumb lines of C7; TK, thoracic kyphosis; LL, lumbar lordosis; PI, pelvic incidence; PT, pelvic tilt; SS, sacral slope.

### Possible factors affecting the postoperative CSA

We used univariate correlation analysis to determine if there were any possible factors that influenced the postoperative C2-7 angle, so that the mean postoperative TK, T10-L2 kyphosis and LL were significantly correlated with the mean postoperative C2-7 angle, respectively (TK, *r* = -0.379; *p* = 0.043; *r* = 0.387, T10-L2 kyphosis, *p* = 0.034; *r* = -0.384; LL, *p* = 0.036). None of the other factors examined were significantly correlated, as shown by the *p*-values (Table 4).

**Table 4:** Univariate correlation analyses of 3-year postoperative C2-7 angle.

	Characteristic	*r*	*p* value
	FV	0.315	0.319
**Postoperative spinal sagittal parameters at the 3-year post-operation**	SVA (C7PL)	-0.131	0.499
	TK	-0.379	0.043*
	T10-L2 kyphosis	0.387	0.034*
	LL	-0.384	0.036*
	PI	-0.181	0.428
	PT	0.047	0.823
	SS	0.054	0.837

FV, fused vertebrae; SVA, sagittal vertical axis; C7PL, plumb lines of C7; TK, thoracic kyphosis; LL, lumbar lordosis; PI, pelvic incidence; PT, pelvic tilt; SS, sacral slope

### Comparison of clinical outcome and assessment

None of the patients in the present study complained of neck or low back pain during the period up to 3 years after surgery. Each of the domains of SRS-22 or ODI score were not changed at 1-year and 3-year post-operation compared to pre-operation, and no obvious statistically significant difference was found (Table 5).

**Table 5:** Comparison of preoperative, 1-year postoperative, and 3-year postoperative clinical outcomes (SRS-22 and ODI questionnaire) among all cases.

	Pre-operation	1-year post-operation	3-year post-operation	*p* value (Pre- vs 1-year post-)	*p* valu e (Pre- vs 3-year post-)	*p* value (1-year- vs 3-year post-)
**SRS-22 domains** Function Pain Self-image Mental health Satisfaction Total	3.7 ± 0.2 4.7 ± 0.3 3.2 ± 0.6 3.6 ± 0.2 3.9 ± 1.3 4.4 ± 0.1	3.6 ± 0.5 4.7 ± 0.1 3.3 ± 0.7 3.6 ± 0.3 4.0 ± 0.1 4.5 ± 0.4	3.6 ± 0.2 4.8 ± 0.2 3.5 ± 0.5 3.7 ± 0.2 4.0 ± 0.8 4.6 ± 0.2	0.781 0.852 0.508 0.901 0.344 0.303	0.855 0.603 0.155 0.889 0.214 0.185	0.867 0.667 0.147 0.801 0.778 0.294
**ODI score (%)**	8.4 ± 9.8	6.2 ± 4.5	5.8 ± 3.7	0.402	0.205	0.373

Values indicate mean ± standard deviation.

SRS, scoliosis research society; ODI, oswestry disability index.

## Discussion

In the present study, we showed that changes in thoracic and lumbar sagittal alignment influenced CSA after correction surgery in Lenke type 6 AIS patients, resulting in a tendency toward reduction of cervical kyphosis. On the contrary, the magnitude of coronal curve correction did not affect each individual spinal sagittal parameter. The findings of this study have important implications for evaluating the influence of correction surgery on CSA because only a few studies have investigated the association between CSA and other sagittal alignments before and after correction surgery in patients with Lenke type 6 AIS.

To date, many reports have investigated changes in various spinal sagittal parameters after correction surgery in AIS patients [[14], [15], [16],[20], [21], [22]]. Wang et al. summarized that preoperative CSA was mainly associated with thoracic and lumbar sagittal alignment in each type of curve of AIS patients (Lenke type 1-6) [13]. Cho et al. showed that C2-7 angle and C2-7 SVA improved postoperatively [15]. Noteworthy, Yagi et al. indicated that the CSA of AIS patients was closely related to the global sagittal spine balance rather than TK [12]. However, most of these studies have included cases with a major thoracic curve, such as Lenke type 1, and a few cases of Lenke type 6 AIS were included. Therefore, no study has ever clearly indicated the relationship between CSA and other sagittal alignments after surgery in strictly only Lenke type 6 AIS patients, and it is not yet clear what factors affect postoperative CSA. Thus, this study is the first report that examined in detail whether CSA on radiographic findings was affected by changes in other sagittal alignments after correction surgery by comparing pre-operation and 3-year post-operation, in patients with Lenke type 6 AIS.

In the present study, the mean cervical lordosis was -4.5 ± 9.7°, T1 slope 14.1 ± 5.9°, TK 13.9 ± 8.0°, T10-L2 angle 1.4 ± 3.5°, LL 43.5 ± 6.7°, SS 34.8 ± 6.7°, and PT 15.4 ± 3.3° after correction surgery. Recently, Lee CS et al. examined the whole-spine radiographsto determine the "normal" radiographic parameters of the sagittal profile of the spine in 181 asymptomatic children. And they reported that the mean cervical lordosis was 4.8 ± 12.0°, TK 33.2 ± 9.0°, T10-L2 angle 5.6 ± 8.4°, LL 48.8 ± 9.0°, SS 34.9 ± 6.6°, and PT 9.4 ± 6.1° [23]. In addition, Hiyama et al. analyzed the characteristics of sagittal alignment, including the cervical spine, in AIS patients with a single thoracic curve (Lenke type 1) compared with the age-matched normal population. They showed that the mean cervical lordosis was 2.5 ± 15.0°, T1 slope 17.8 ± 7.9°, TK 21.3 ± 7.6°, LL 40.9 ± 11.8°, and SS 28.5 ± 8.3° in the control (asymptomatic children) group [24]. Considering based on the results of these reports, the sagittal parameters of the thoracic and lumbosacral spine, which was the correction and fixation area, approached the above previous data. However, for the cervical spine, lordotic effect was observed, although it still did not approximate to them in this study.

The association between postoperative changes in CSA and the location of UIV has been still controversial in Lenke type 6 AIS patients [12,13]. Yanik et al. showed that the mean cervical lordosis and TK were significantly decreased after correction surgery at 2-year follow-up period in Lenke type 3 and 6 AIS patients. These authors also summarized that CSA was found to be independent of the UIV level postoperatively, but it was mainly influenced by T1 slope and TK [16]. Conversely, the present study demonstrated that C2-7 angle and T1 slope increased significantly with changes in TK after correction surgery in 44 patients with Lenke type 6 AIS. This difference might be related to the location of the UIV before surgery. Yanik et al. selected the level of UIV between T2 and T4 according to the preoperative shoulder balance, while we determined the level of UIV according to the UEV of the minor curve. In fact, most of the patients whose UIV was located at T5 or lower levels, and only three patients whose UIV was located at T4 or higher levels were included in this study. Meanwhile, in other curve types of AIS patients, Ketenci et al. reported that C2-7 angle and T1 slope significantly decreased postoperatively in Lenke type 1 AIS patients whose UIV was located at T2 or T3. They also suggested that extending the fusion to upper levels may cause proximal thoracic hypokyphosis, which can lead to loss of cervical lordosis [25]. Legarreta et al. demonstrated that UIV at T4 or lower levels had a lordotic effect on CSA in Lenke type 1 AIS patients postoperatively, whereas a kyphotic effect was observed with UIV at T3 or above [26]. Furthermore, we previously showed that CSA was influenced by changes in thoracic kyphosis following correction surgery in Lenke type 5 AIS patients whose UEV (UIV was selected as the UEV or one-level caudal to the UEV) was located at T9 or higher levels [22]. Unfortunately, the results of the present study cannot be compared directly to those of previous studies, however, which level selected for UIV from the upper to middle thoracic spine may affect lordotic or kyphotic change on the CSA after correction surgery in Lenke type 6 AIS patients.

**Fig. 1:**
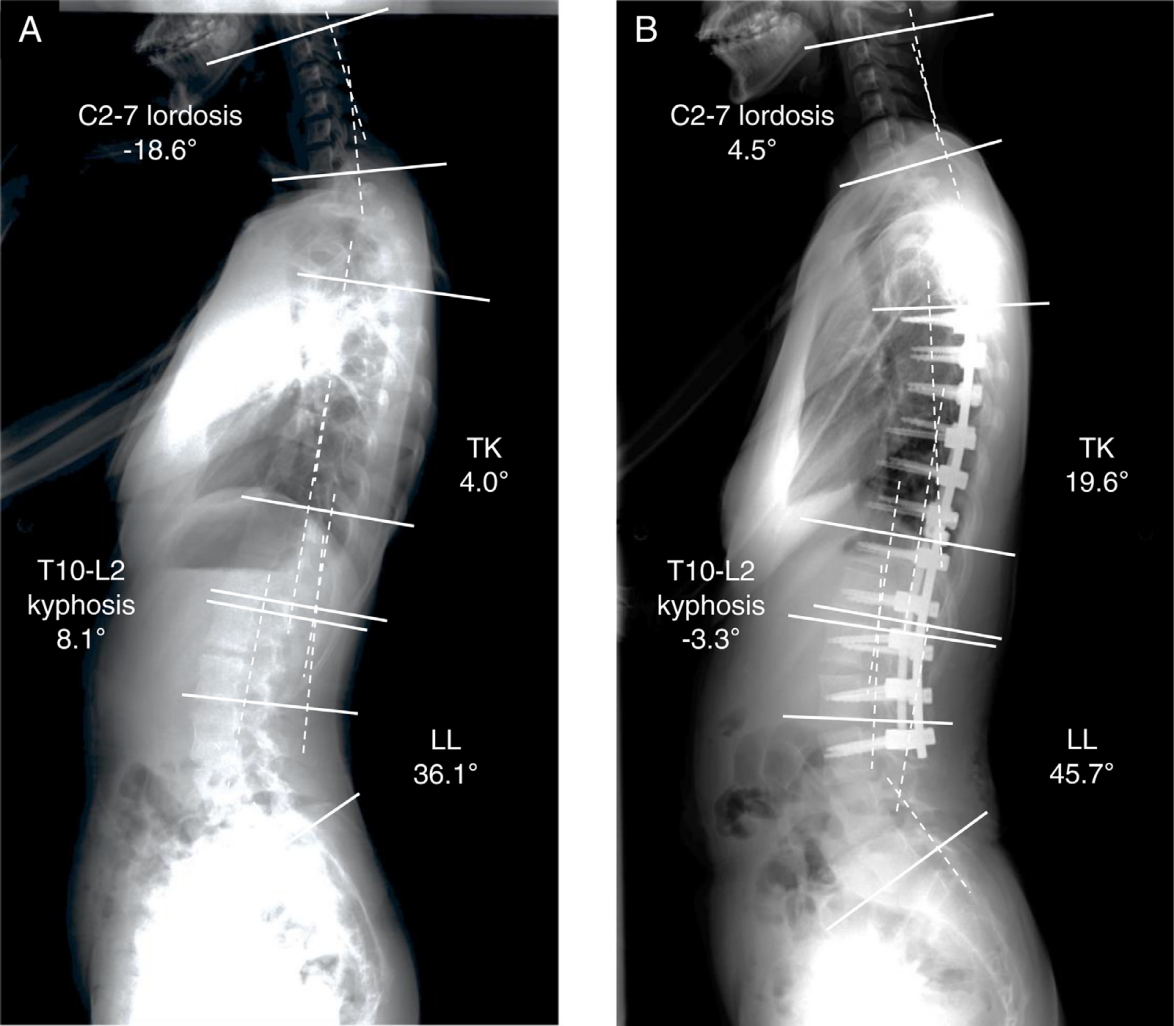
Representative pre- and 3-year postoperative whole spine standing sagittal radiographs for the patients with Lenke type 6 adolescent idiopathic scoliosis. After selective posterior correction and fusion surgery with pedicle screws from T5 to L3, each of the spinal sagittal alignments were corrected at a 3-year follow-up period. Preoperative C2-7 lordosis (C2-7 Cobb angle) of -18.6°, TK of 4.0°, T10-L2 kyphosis of 8.1°, and LL of 36.1°. Whereas, at 3-year post-operation, C2-7 lordosis of 4.5°, TK of 19.6°, T10-L2 kyphosis of -3.3°, and LL of 45.7°.

TK, thoracic kyphosis; LL, lumbar lordosis

Although several reports indicated that cervical sagittal malalignment could influence the development of neurological symptoms with spinal cord compression in older age in AIS patients, it remains controversial whether postoperative changes in CSA would affect clinical symptoms and HRQOL outcomes [6,7,27]. Ames et al. reported that cervical sagittal curvature is related to various spinal sagittal parameters that may influence neck pain and HRQOL [28]. Youn et al. found that there was a significant relationship between postoperative changes in CSA and HRQOL outcomes, such as SRS-22 and Short Form-36 (SF-36), in patients with AIS [7]. In contrast, Chang et al. showed that the SRS-22 score at the final follow-up did not differ significantly between Lenke type 5 and 6 AIS patients who underwent selective thoracolumbar-lumbar curve fusion [29]. In the current study, similarly, each domain of the SRS-22 questionnaire and ODI score did not change significantly at 3 years postoperatively. However, in Lenke type 6 AIS, the long-term results of the relationships between cervical sagittal parameters and neck pain, SRS-22, ODI, age-related degeneration (disc degeneration, spinal compression, and post-junctional kyphosis), etc., are not yet well known (Fig. 1). Therefore, future investigations on these clinical correlates would be useful to validate our findings in this study and identify the real benefits of correction surgery for Lenke type 6 AIS patients, which will constitute the topic of a further prospective study.

Our present study has several significant limitations that should be noted. First, this study had a retrospective design, which inevitably lowered the evidence level. Second, the sample size was relatively small, and the statistical power was not strong enough to draw conclusions about the precise clinical outcomes for Lenke type 6 AIS patients. Finally, standard standing erect whole-spine posteroanterior and lateral radiographs were used, which are suboptimal as measures of rotational deformity. In addition, although we have instructed the patients to look straight ahead as firmly as possible, various cervical sagittal parameters could be influenced by slight changes in the position of the patient's head. Despite these limitations, the present study provides important results on the presence of CSA changes in Lenke type 6 AIS patients after correction surgery. The major interest of this study is constituted by unprecedented analyses that focus on the influence of the magnitude of coronal curve correction or the relationship between postoperative CSA and other sagittal profiles in Lenke type 6 AIS patients.

## Conclusion

Here, we examined the variations in CSA and any possible factors that affect them following correction surgery in Lenke type 6 AIS patients. CSA was influenced by the improvement of the thoracic and lumbar sagittal profiles, not the correction for coronal deformity after correction surgery with pedicle screw constructs. However, because the number of cases was relatively small in this study, future studies with a larger sample size will be warranted to bring more precise conclusions.

## Declaration of Competing Interest

The authors declare that they have no known competing financial interests or personal relationships that could have appeared to influence the work reported in this paper.
